# Atypical Presentations of Neuroleptic Malignant Syndrome: Toward a Redefinition of Diagnostic Criteria?

**DOI:** 10.1192/j.eurpsy.2026.11259

**Published:** 2026-07-31

**Authors:** I. Bouamoud, S. Belbachir, A. Ouanass

**Affiliations:** Psychiatry, Arrazi Hospital, Sale, Morocco

## Abstract

**Introduction:**

Neuroleptic Malignant Syndrome (NMS) is a rare but severe reaction to dopamine antagonists, typically characterized by hyperthermia, muscle rigidity, altered mental status, and autonomic instability. However, increasing reports of atypical or incomplete presentations, especially with second-generation antipsychotics, challenge the applicability of traditional diagnostic criteria.

**Objectives:**

To describe the atypical clinical presentations of NMS observed in a recent cohort.

To evaluate the sensitivity of different diagnostic criteria (DSM-5, Levenson, Pope, Caroff ).

To propose the need for a more inclusive and flexible diagnostic framework for NMS.

**Methods:**

We conducted a retrospective case-control study at Arrazi University Psychiatric Hospital (Salé, Morocco) from 2020 to 2023.

- **Cases:** 43 patients diagnosed with NMS based on DSM-5 criteria.
- **Controls:** 86 hospitalized patients receiving neuroleptics but without NMS.
- Data collection included sociodemographic, clinical, biological, and therapeutic variables.
- Diagnostic compatibility with various international criteria was assessed.

**Results:**

Among the 43 confirmed NMS cases, only **DSM-5 criteria** identified all patients (100%), while older criteria were less sensitive: **Levenson (19%)**, **Pope (11.6%)**, and **Caroff (4.7%)**.

**Muscle rigidity** (79.1%) and **altered mental status** (51.2%) were the most frequent symptoms, while **hyperthermia** appeared in only **18.6%** of cases.

**CPK elevation** was found in **97.7%** of patients. Multivariate analysis revealed two significant risk factors: **haloperidol use** (OR = 16.46) and **prior NMS history** (OR = 62.07).

These findings highlight the **frequent atypical presentations** of NMS and the **limitations of traditional diagnostic criteria**.

**Conclusions:**

Our findings confirm a growing trend of **atypical or incomplete forms of NMS**, which are frequently underdiagnosed using classical criteria. The **DSM-5 criteria** proved to be the most inclusive, but even they may overlook subtle early presentations. These results support the urgent need to **revise and modernize diagnostic approaches** to NMS, especially in psychiatric settings where early intervention can prevent serious complications.

**Disclosure of Interest:**

None Declared

